# Neddylation status determines the therapeutic sensitivity of tyrosine kinase inhibitors in chronic myeloid leukemia

**DOI:** 10.1038/s41598-025-04153-7

**Published:** 2025-05-30

**Authors:** Congyi Zhang, Yikai Yao, Qiuting Qian, Xiongyu Han, Yunkun Lu, Xinyi Jiang, Hongqiang Cheng, Xue Zhang, Ying Chi, Yuehai Ke, Peng Xiao

**Affiliations:** 1 Department of Pathology and Pathophysiology, and Department of Respiratory Medicine at Sir Run Run Shaw Hospital, Zhejiang University School of Medicine, Hangzhou, China; 2 Zhejiang University-University of Edinburgh Joint Institute, ZJU Haining International Campus, Jiaxing, China; 3 Sir Run Run Shaw Hospital, Zhejiang University School of Medicine, Hangzhou, China; 4 Institute of Immunology, Zhejiang University School of Medicine, Hangzhou, China

**Keywords:** Chronic myeloid leukemia, Tyrosine kinase inhibitor, Neddylation, Post-translational modification, Resistance, Cancer, Cell biology

## Abstract

BCR::ABL1-targeting tyrosine kinase inhibitors (TKIs) dominate the treatment of chronic myeloid leukemia (CML) over the past decades. In this study, we reported an unexpected role of neddylation inhibitors in desensitizing the therapeutic efficacy of BCR::ABL1-targeting TKIs in CML. Unlike their function in reducing drug resistance in many solid tumors, we revealed that neddylation inhibitors counteracted the cytotoxicity of TKIs against CML cells, both in cellular experiments and in animal model. Conversely, neddylation agonist sensitized the function of TKIs. RNA sequencing data revealed that neddylation inhibitor reversed the transcriptomic changes induced by TKI. Co-immunoprecipitation (co-IP) assay identified ABL1 kinase domain as a novel substrate for neddylation. Furthermore, an artificial intelligence (AI) 3-Dimensional spatial structure binding technology was employed to predict the impact of neddylation on the structure of ABL1 kinase domain. Finally, we provided potential evidence showing that TKI therapy decreased the expression of neddylation enzymes in the bone marrow of CML patients. Hence, our study offers new insights into the post-translational modification (PTM)-mediated drug resistance, and highlights the potential clinical benefits of neddylation agonists in improving the responsiveness of BCR::ABL1 TKIs in CML.

**Supplementary Information:**

The online version contains supplementary material available at 10.1038/s41598-025-04153-7.

## Introduction

The primary etiological factor of chronic myeloid leukemia (CML) is the BCR::ABL1 fusion protein, which is a highly active tyrosine kinase capable of initiating multiple downstream oncogenic pathways^1,2^. Although the development of tyrosine kinase inhibitors (TKIs) targeting BCR::ABL1 has significantly revolutionized the treatment of CML, resistance to TKIs eventually occurs in a portion of CML patients, leading to therapy failure and further progression of CML^3^. Thus, overcoming TKI resistance represents one of the top challenges in the management of CML.

Traditionally, TKI resistance is commonly attributed to specific point mutations that alter the drug’s binding affinity through changing protein conformation^4,5^. Regrettably, mutations occur at the genetic level and thus are basically irreversible. Post-translational modifications (PTMs) represent another pivotal event that have profound influence on protein conformation^6^. In contrast to genetic mutations, most types of PTMs are reversible, rendering them ideal targets for the development of anti-cancer agents^7^. Neddylation is a reversible covalent conjugation of an ubiquitin-like molecule NEDD8 to substrate proteins^8^. Neddylation of specific proteins typically does not lead to proteasome degradation. Instead, it influences protein stability, conformation, or subcellular localization, through which neddylation modification plays essential roles in determining cell functions^9^. In recent years, emerging studies have revealed that hyper-neddylation in solid tumor cells promotes their malignant behaviors and contributes to drug resistance^10–12^. Therefore, neddylation inhibitors have been tested in various clinical trials for the treatment of cancer or the mitigation of drug resistance^13–15^. Our group has identified a classical oncoprotein - SHP2, as a novel substrate of neddylation. The deneddylation of SHP2 drives tumor immunosuppression by regulating CD47/SIRPα signal^16^. However, to date, the roles of neddylation on the treatment of hematological malignancies remain elusive.

## Results

### Hyponeddylation desensitizes the function of BCR::ABL1-targeting TKIs

**Fig. 1:**
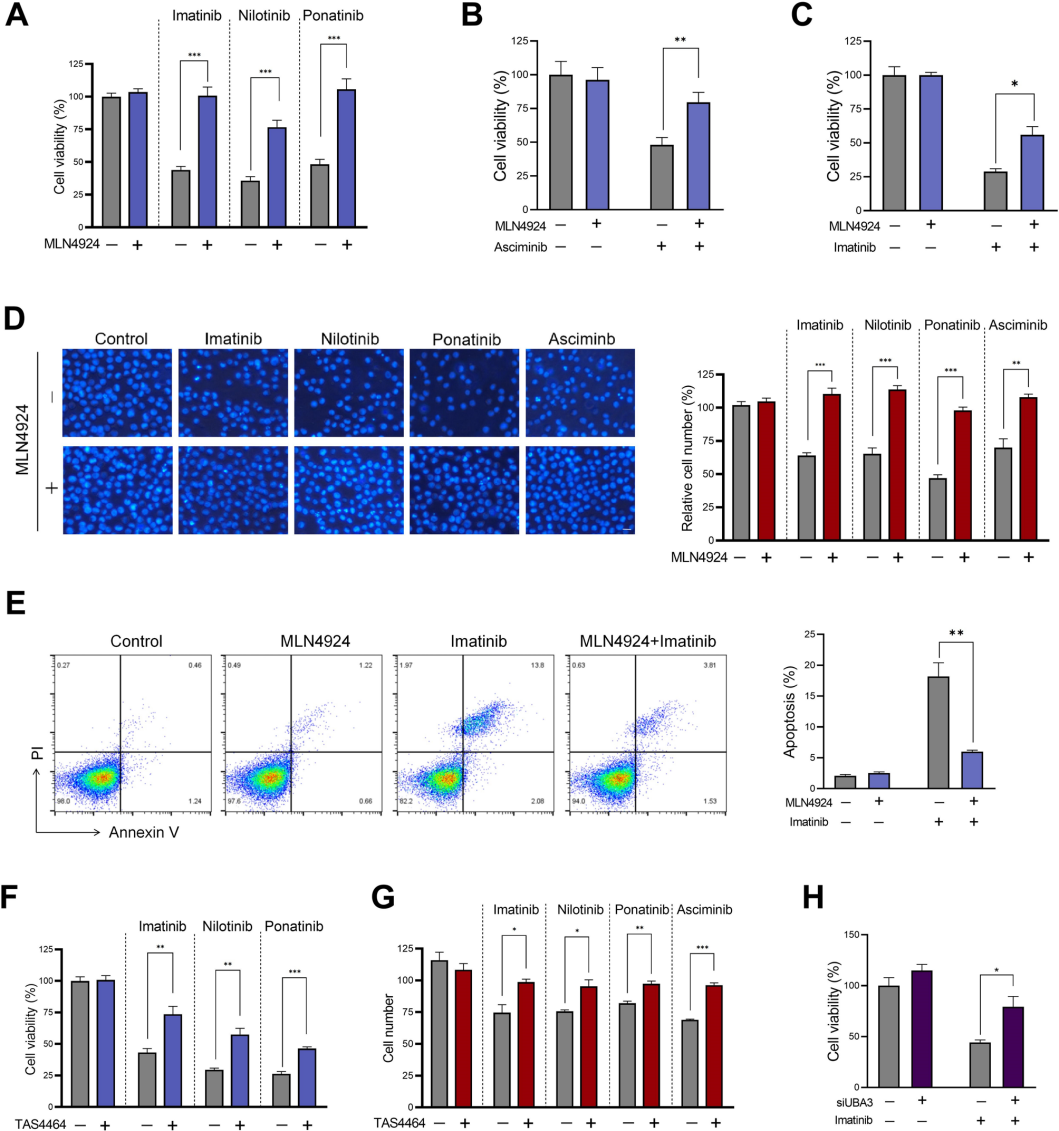
Neddylation inhibitor blunted the therapeutic function of BCR::ABL1-targeting TKIs. (A, B) K562 cells were treated with imatinib (0.5 µM), nilotinib (0.1 µM), ponatinib (2.5 nM) (**A**), or asciminib (50 nM) (**B**) in the presence or absence of MLN4924 (25 µM) for 24 h. Cell viability was evaluated by CCK8. (**C**) KU812 cells were treated with imatinib in the presence or absence of MLN4924 for 24 h. Cell viability was evaluated by CCK8. (**D**) K562 cells were treated with the indicated TKIs in the presence or absence of MLN4924 (25 µM) for 24 h, cell counting was performed following Hoechst staining. (**E**) K562 cells were treated with imatinib (0.5 µM) in the presence or absence of MLN4924 (25 µM) for 24 h. Cell apoptosis was evaluated by Annexin V/PI staining. (**F**) K562 cells were treated with imatinib (0.5 µM), nilotinib (0.1 µM), or ponatinib (2.5 nM) in the presence or absence of TAS4464 (1 µM) for 24 h. Cell viability was evaluated by CCK8. (**G**) K562 cells were treated with the indicated TKIs in the presence or absence of TAS4464 (1 µM) for 24 h, cell counting was performed following Hoechst staining. (**H**) UBA3 was silenced in K562 cells, followed by imatinib (0.5 µM) treatment for 24 h, cell viability was evaluated by CCK8. Unpaired, two-tailed Student’s t-test. *p < 0.05; **p < 0.01; ***p < 0.001.

Firstly, in an effort to investigate the impact of neddylation modification on the therapeutic effectiveness of BCR::ABL1-targeting TKIs in CML, we treated K562 CML cells with three different TKIs, including imatinib (the first-generation TKI), nilotinib (a second-generation TKI), and ponatinib (a third-generation TKI), which are designed to target BCR::ABL1 carrying different mutation sites. These TKIs were either used alone or in combination with MLN4924, a neddylation inhibitor which suppresses the activity of NEDD8 activating E1 enzyme^13^. K562 cells are known to be resistant to MLN4924^17^, we used an MLN4924 concentration that did not cause discernible cytotoxicity to K562 cells as determined by our preliminary IC50 test. Unexpectedly, contrary to its function in mitigating drug resistance in many previous studies^15,18,19^, neddylation inhibition by MLN4924 largely counteracted, but not sensitized the therapeutic effect of TKIs in CML cells. In the presence of MLN4924, all three TKIs failed to induce obvious cytotoxic effect in K562 cells (Figure 1 A). The above three TKIs inhibit the activity of BCR::ABL1 by targeting its ATP-binding sites. Non-constitutively active ABL1 undergoes conformational changes between active and inactive states, this led to the development of the fourth-generation BCR::ABL1 TKI - asciminib, which allosterically targets BCR::ABL1 through a non-ATP competitive conformational mechanism. We observed that the cytotoxicity of asciminib was also largely lost when used in combination with MLN4924 (Figure 1B). Besides K562 cells, MLN4924 reversed the inhibitory effect of imatinib on another CML cell line, KU812 as well (Figure 1 C). The capacity of MLN4924 in antagonizing TKI function was further confirmed by cell counting (Figure 1D) and cell apoptosis assay (Figure 1E). Next, we employed another neddylation inhibitor, TAS4464. Similar to MLN4924, TAS4464 efficiently reversed the cytotoxicity of multiple TKIs against K562 cells (Figure 1 F, G). Furthermore, we silenced UBA3 - a subunit of neddylation E1, in K562 cells, and found that UBA3 silencing recapitulated the effect of neddylation inhibitors in terms of reversing the cytotoxicity of TKI (Figure 1H).

**Fig. 2:**
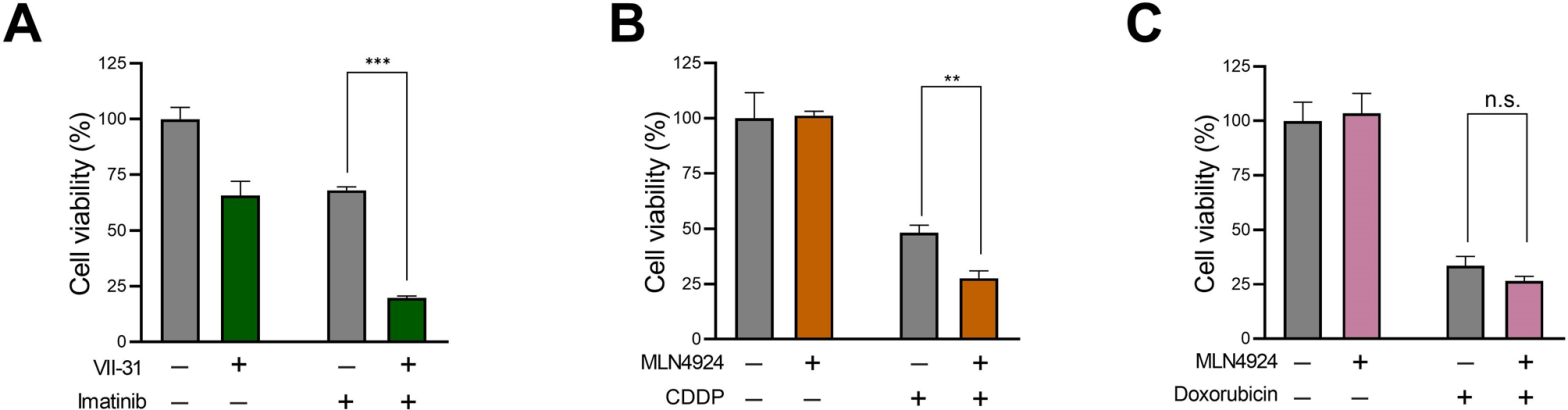
Neddylation activator sensitizes the function of imatinib. (**A**) K562 cells were treated with imatinib (0.1 µM) in the presence or absence of VII-31 (80 nM) for 24 h. Cell viability was evaluated by CCK8. (B, C) K562 cells were treated with 10 µM cisplatin (CDDP) (**B**) or 0.5 µM doxorubicin (**C**) in the presence or absence of MLN4924 (25 µM) for 24 h. Cell viability was evaluated by CCK8. Data are presented as mean ± SEM. Unpaired, two-tailed Student’s t-test. *p < 0.05; **p < 0.01; ***p < 0.001.

To further validate the impact of neddylation status on the function of TKI, we introduced VII-31, a novel neddylation activator^20^. In contrast to MLN4924, a notable synergistic cytotoxic effect was observed between VII-31 and imatinib (Fig. 2A), indicating that increasing neddylation can sensitize the function of TKI. On the other hand, in order to examine the impact of neddylation on non-BCR::ABL1-targeting anti-tumor drugs, two chemotherapeutic agents, cisplatin and doxorubicin, were used. Unlike its effect on TKIs, MLN4924 did not attenuate the cytotoxic effects of cisplatin or doxorubicin (Fig. 2B, C). Contrarily, MLN4924 sensitized the function of CDDP, indicating that the drug resistance effect of MLN4924 was specific to BCR::ABL1-targeting TKIs in CML cells.

## Deneddylation reverses imatinib-induced transcriptomal changes in CML cells

**Fig. 3:**
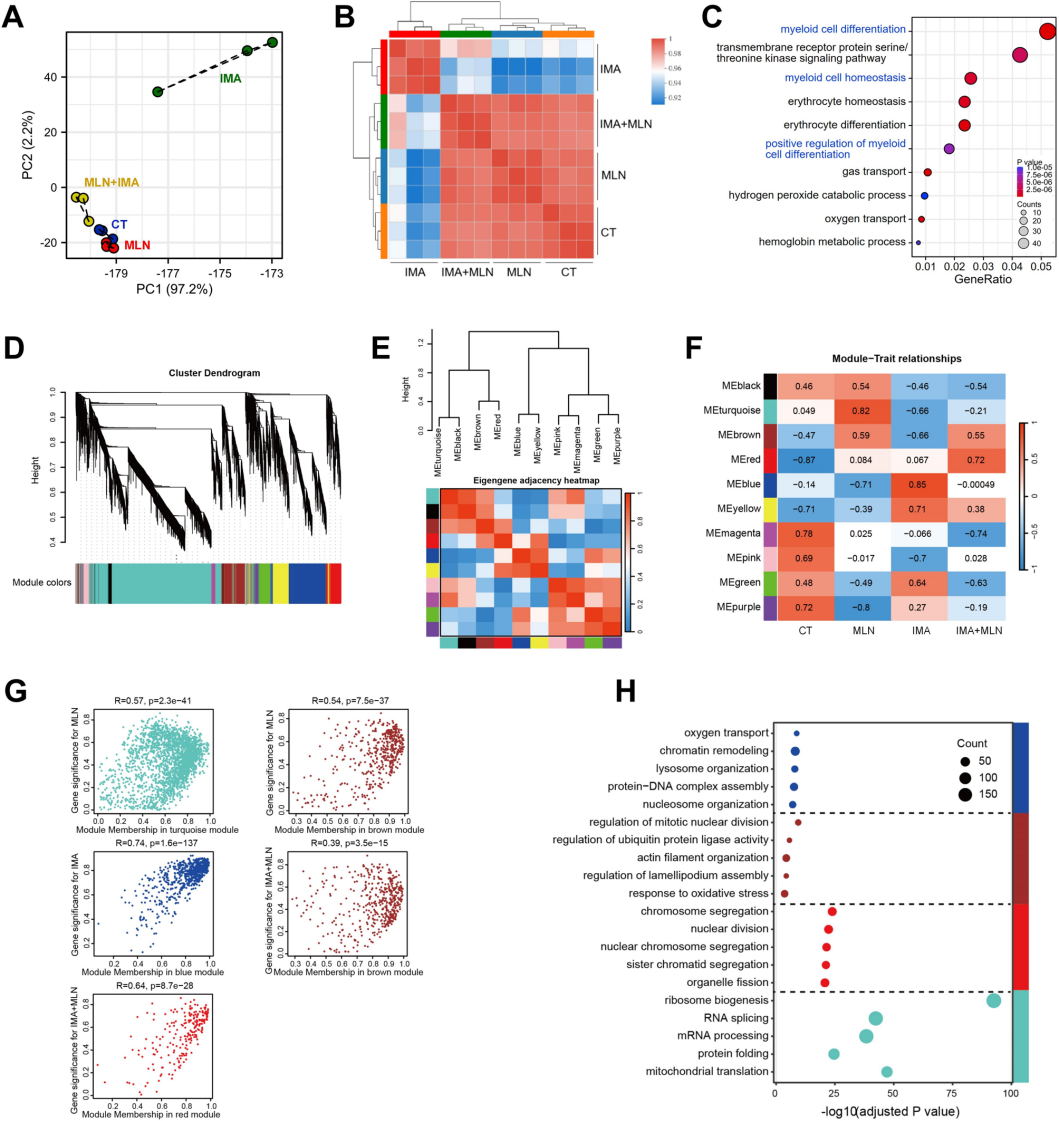
RNA sequencing analysis of the transcriptomal features in CML cells. (A, B) K562 cells were treated with imatinib (0.5 µM) in the presence or absence of MLN4924 (25 µM) for 12 h, RNA sequencing was performed. PCA analysis (**A**) and sample correlation analysis (**B**) were performed. (**C**) GO analysis of the DEGs between imatinib group and imatinib + MLN4924 group. (D-H) Construction of WGCNA network of all RNA sequencing samples: (**D**) Dendrogram of gene topological matrix branch. (**E**) Dendrogram and correlation heatmap of 10 eigengenes in each module. (**F**) Heatmap of correlations between gene modules and each group of RNA-seq samples. The Pearson correlation coefficient (R) between different gene modules and clinical traits were shown in the heatmap. (**G**) Scatter plot of gene significance for selected groups with module membership (MM) in the most significantly positively correlated module. The brown module showed significant positive correlations with both MLN and IMA + MLN groups. (**H**) GO enrichment analysis of genes in blue, red, turquoise, and brown modules.

Subsequently, RNA sequencing was conducted to look at the drug’s impact on the transcriptome of K562 cells. Principal Component Analysis (PCA) showed that imatinib-treated K562 cells were clearly separated from control cells, while this separation was largely by reversed by the simultaneous MLN4924 treatment (Fig. 3A). In addition, the transcriptome of imatinib + MLN4924-treated K562 cells exhibited a stronger correlation with control cells rather than imatinib-treated cells (Fig. 3B), indicating that neddylation inhibition counteracts the impact of imatinib on the transcriptome of CML cells. Gene Ontology (GO) analysis showed that the differentially-expressed genes (DEGs) between imatinib group and imatinib + MLN4924 group were highly enriched in pathways related to the differentiation and function of myeloid cells (Fig. 3C).

We further conducted Weighted Gene Co-Expression Network Analysis (WGCNA) on the top 5,000 genes that showed the most significant expression differences across all RNA-seq samples. We first determined the soft threshold β = 12 to construct the co-expression network (Supplementary Figure S1A-C). After selecting the soft threshold, we identified 10 gene modules, each containing 50 to 2000 genes with significant correlations within all RNA-seq samples (Fig. 3D). Next, Pearson correlation coefficients were calculated to assess the similarity between gene modules. It was found that the differentiation between gene modules was good, indicating the correctness of gene modules (Fig. 3E).Then we found that the turquoise, blue, and red modules showed the most significant positive correlations with MLN, IMA, and IMA + MLN group, respectively, while the brown module showed significant positive correlations with both MLN and IMA + MLN (Fig. 3F), indicating that the genes in brown module may be involved in causing IMA resistance after adding MLN. The feature genes in all these modules (turquoise, blue, red, and brown) showed a significant positive correlation between gene significance in each group and module membership of each gene, suggesting that the WGCNA analysis identified important gene sets significantly associated with these groups, providing potential candidates for further investigation (Fig. 3G). GO enrichment analysis revealed that genes of the brown module were enriched in functions such as regulation of ubiquitin protein ligase activity, cell migration, and oxidative stress related terms. Meanwhile, genes in the red module were enriched in cell cycle-related terms (Fig. 3H). Therefore, deneddylation counteracts the transcriptional changes in imatinib-challenged CML cells.

## Neddylation modification leads to structural change of ABL1

**Fig. 4:**
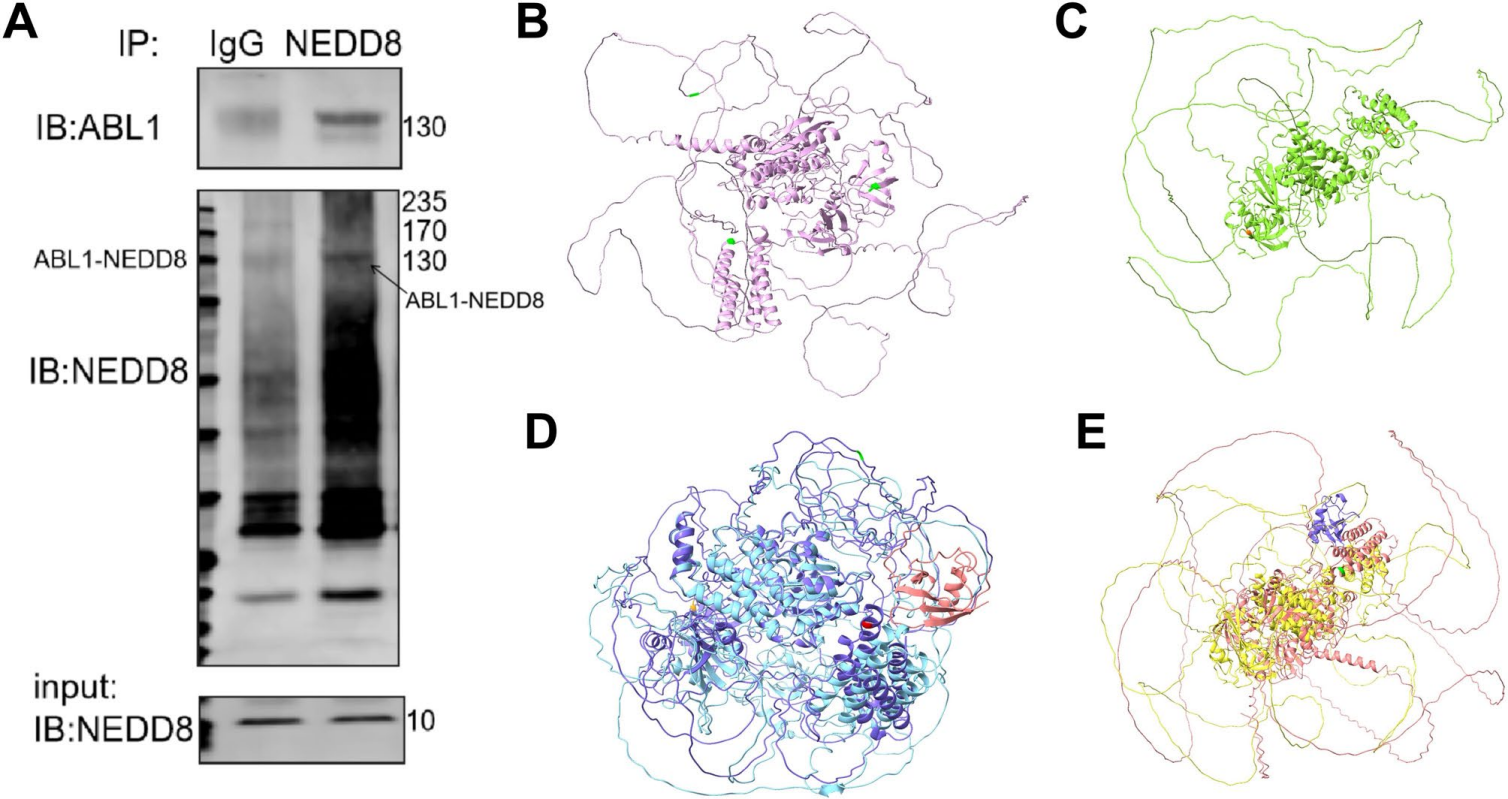
ABL1 is a novel neddylation substrate. (**A**) The binding of NEDD8 to ABL1 domain was evaluated by co-IP in K562 cells. (**B**) ABL1 structure predicted by an AI large-molecule structure predicting tool ESM-fold. The light-green sites represent K1080, K87, and K756. (**C**) ABL1 structure predicted by AlphaFold3. The orange sites represent K1080, K87, and K756. (**D**) ABL1 structure before and after deneddylation as predicted by ESM-fold. The blue color represents structure after deneddylation of ABL1, the purple color indicates ABL1 bound to a NEDD8 molecule. The pink color represents the NEDD8 molecule. The red site is K1080, the yellow site is K87, the green site is K756. (**E**) ABL1 structure before and after deneddylation as predicted by AlphaFold3. The yellow color indicates ABL1 structure after deneddylation, the pink color represents ABL1 neddylated by one NEDD8 molecule. The purple color represents NEDD8.

The aforementioned findings prompted us to further explore whether BCR::ABL1 could serve as a direct target for neddylation. Since the conformation of ABL1 kinase domain determines the activity of BCR::ABL1 fusion protein, it serves as a direct target of BCR::ABL1 TKIs. Therefore, the structural analysis of ABL1 kinase domain was used to design drugs^21^. Through performing co-immunoprecipitation (co-IP) experiment using anti-NEDD8 antibody, ABL1 was confirmed to be co-precipitated with NEDD8 (Fig. 4A). Therefore, we identified ABL1 as a novel neddylation substrate.

Non-Cullin substrates usually have multiple neddylation sites, which are broadly dispersed across the exposed lysine residues on the target surface. The ABL1 kinase domain contains 82 lysine residues that are potential substrates for neddylation. By using the Neddypreddy Prediction Platform^22^, K1080, K87, and K756 were identified as the most probable sites on ABL1 (Supplementary Table 1). Therefore, we next employed the artificial intelligence (AI) 3-Dimensional spatial structure binding technology, combining the known structure of two ABL1 structure domains deciphered from cryo-EM or X-ray crystallization (we call these golden truth) and other structure domains without golden truth. From the established sequences of the complete ABL1, we predicted this complete molecule structure using both ESM-fold^23^ (Fig. 4B) & AlphaFold3^24^(Fig. 4C), which are the two most popular protein multimer structure prediction tools. As shown in Fig. 3B and C, the green or orange residues represented K1080, K87, and K756. Additionally, we utilized ESM-fold (Fig. 4D) and AlphaFold3 (Fig. 4E) space structure binding technology to predict the structure of ABL1 before and after deneddylation. It was found that when ABL1 has one and only one neddylation residue, its structure changed from blue to purple. The pink neddylation residue is very close to the most probable neddylation site K1080 in bright red. The orange residue, K87, had the second-highest probability of being a neddylation site, while the bright green residue, K756, ranked the third in probability (Fig. 4D). Results from AlphaFold3 demonstrated that the yellow part indicated the deneddylated ABL1 structure, while the pink part denoted the ABL1 structure upon binding to purple NEDD8, leading to structural alterations. The purple NEDD8 is very close to the most probable neddylation site K1080 in bright green. (Fig. 4E). Furthermore, we conducted a comparison between AlphaFold3 and ESM-fold prediction results. By comparing the known golden truth, AlphaFold3 accuracy was slightly higher than ESM-fold. From an alternative perspective, the results from AlphaFold3 and ESM-fold are similar. Specifically, the structure of the one-NEDD8 molecule is in close proximity to the K1080 residue as anticipated. Therefore, both AlphaFold3 and ESM-fold predict an obvious structural alteration of ABL1 after deneddylation.

## Hyponeddylation drives therapeutic resistance to Imatinib in murine xenograft CML model

**Fig. 5:**
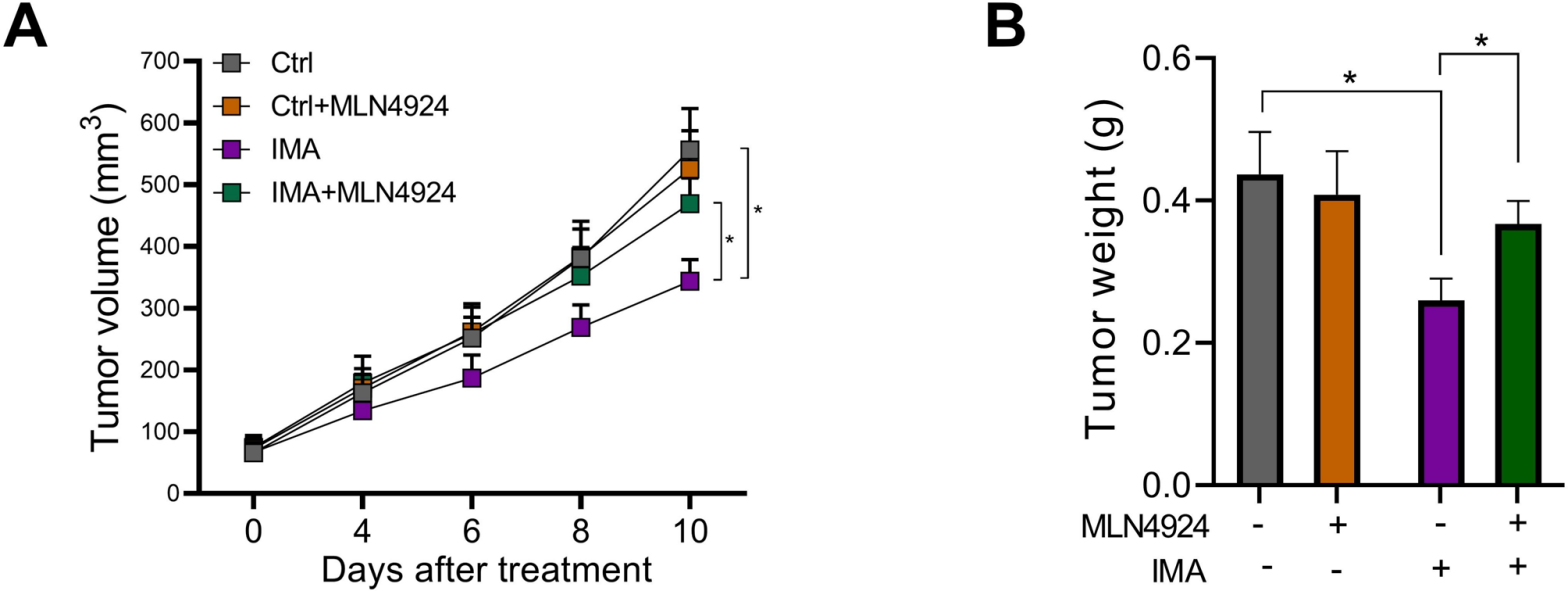
A low neddylation status inhibits the effectiveness of imatinib therapy in murine CML model. (A, B) K562 cells were subcutaneously inoculated into nude mice (*n* = 6/group), followed by the administration of imatinib (100 mg/kg), MLN4924 (50 mg/kg), or imatinib plus MLN4924, tumor growth was monitored (**A**), tumor weight was evaluated at the experimental endpoint (**B**). Data are presented as mean ± SEM. Unpaired, two-tailed Student’s t-test. *p < 0.05.

Next, in an effort to further assess the impact of MLN4924 on the therapeutic sensitivity of imatinib in vivo, we inoculated K562 cells into nude mice, followed by the administration of imatinib, MLN4924, either alone or in combination. As shown in Fig. 5A and B, imatinib effectively suppressed tumor growth and reduced tumor weight. In contrast, the simultaneous administration of MLN4924 blunted the therapeutic effectiveness of imatinib.

**Fig. 6:**
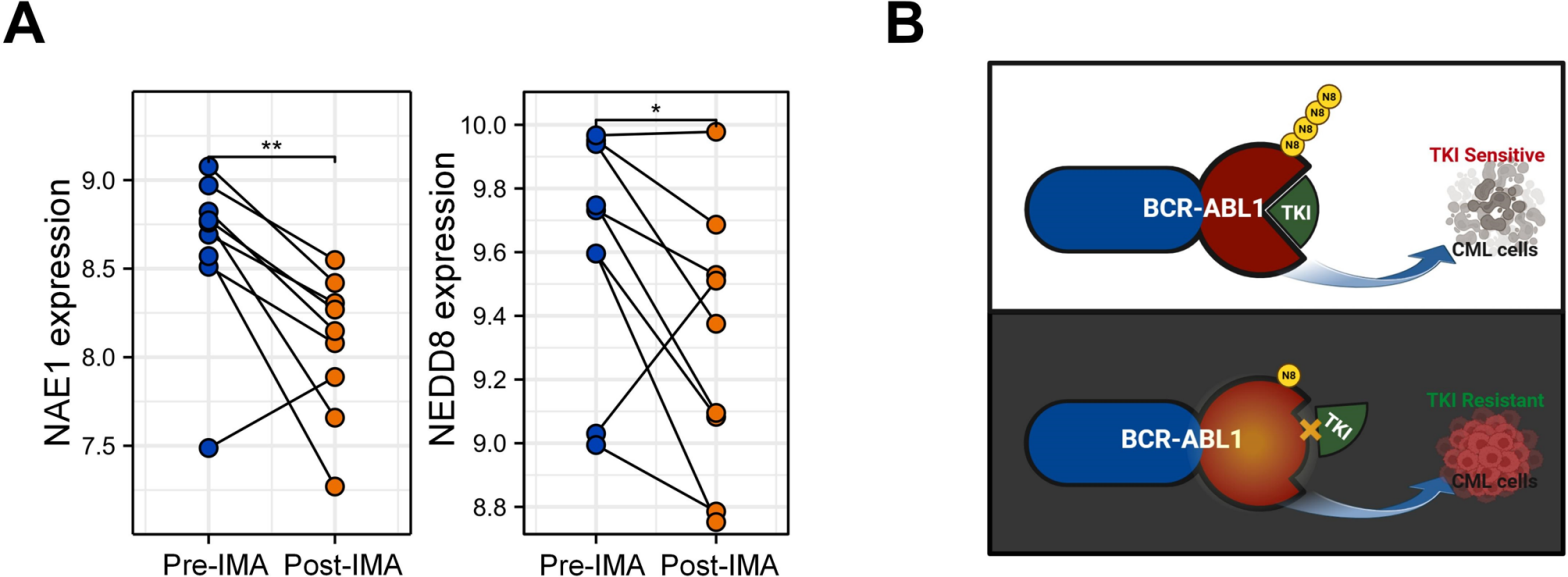
(**A**) The expression of NAE1 and NEDD8 was analyzed using GEO database (GSE33075). Paired, two-tailed Student’s t-test. *p < 0.05; **p < 0.01. (**B**) Model illustrating the mechanism that neddylation of BCR::ABL1 is required for the therapeutic response of TKIs.

Finally, to explore the potential clinical relevance of neddylation and TKI therapy, we analyzed a Gene Expression Omnibus (GEO) dataset^25^, in which bone marrow samples from CML patients were harvested before and after imatinib treatment. The results showed that the expression of both NAE1 and NEDD8 was significantly downregulated in response to imatinib treatment (Fig. 6A). NAE1, a member of the ubiquitin-activating E1 family, is the target of neddylation inhibitors^26^; while NEDD8 encodes an ubiquitin-like protein essential for neddylation. Therefore, this finding suggests that BCR::ABL1-targeting TKI treatment could potentially downregulate the neddylation status of CML cells, thereby leading to the therapeutic insensitivity in a portion of patients.

In summary, we for the first time discovered that ABL1 protein can undergo neddylation, which is essential for the therapeutic effectiveness of TKIs in CML cells. Inhibition of neddylation leads to the failure of TKI (Fig. 6B).

## Discussion

BCR::ABL1-targeting TKIs represent a typical success of precision medicine in cancer treatment. Unfortunately, drug resistance occurs in a fraction of patients who received BCR::ABL1-targeting TKI therapy^27^. It is estimated that more than 90 types of site mutations exist in the kinase domain of BCR::ABL1, each with varying effects on the structure and kinase activity of the BCR::ABL1 protein, leading to different levels of resistance to TKIs. Consequently, a series of TKIs have been developed to treat CML patients carrying specific BCR::ABL1 mutation^28^. Unlike site mutations which irreversibly occur in the genomic level, epigenetic modifications such as PTMs are typically reversible and also significantly impact protein structure. Neddylation is an ubiquitin-like posttranslational modification that is crucial for maintaining cellular homeostasis under physiological conditions. However, hyperneddylation is observed in various types of tumor cells, contributing to their malignant behaviors or drug resistance^8,29^. Thus, neddylation inhibitors are considered as promising anti-tumor strategies, with their efficacy being evaluated in clinical trials, such as advanced non-small cell lung cancer, multiple myeloma, metastatic melanoma, bile duct cancer, acute myeloid leukaemia, and mesotheliomas^30,31^. Due to its pro-resistance effect of neddylation in solid tumors^15,18,19^, we initially hypothesized that neddylation inhibition would have a synergistic effect with BCR::ABL1-targeting TKIs. To our surprise, neddylation inhibitor counteracted the cytotoxic effects of BCR::ABL1-targeting TKIs, as evidenced by both in vitro assays and animal experiments.

In our study, we selected an MLN4924 concentration that did not cause cytotoxic effect on K562 cells. Actually, when used a high dosage, MLN4924 alone significantly reduced the viability of K562 cells, and thus blunted its impact on the resistance of TKIs. Therefore, the precise role of neddylation in the responsiveness of BCR::ABL1-targeting TKIs is multifactorial and complex. It could be influenced by the baseline neddylation status of cells, their sensitivity to neddylation inhibitors, and the expression or activity of other neddylation substrates. To gain a more profound insight into this question in a clinically relevant context, we aim to collect tumor samples from CML patients, and explore potential correlations between cellular neddylation levels and therapeutic outcomes of BCR::ABL1-targeting TKIs.

Compared to its similar effect on multiple BCR::ABL1-targeting TKIs, neddylation inhibition did not antagonize the cytotoxic function of cisplatin or doxorubicin, suggesting that BCR::ABL1 is a potential functional substrate of neddylation. Indeed, we validated that the ABL1 kinase domain could undergo neddylation modification, which may cause structural changes to ABL1 as predicted by the AI tool. Further investigation is needed to determine the potential impact of neddylation-induced conformational alterations of ABL1 on the binding activity of TKIs.

In summary, our study identifies the ABL1 kinase domain as a novel target for protein neddylation, and expands the current knowledge on the drug resistance mechanisms of BCR::ABL1-targeting TKIs. From a clinical perspective, the administration of neddylation agonist may be a promising strategy for sensitizing the therapeutic effectiveness of TKIs in CML patients. In addition, cellular neddylation status could serve as a potential indicator for predicting the therapeutic outcomes in CML patients who received the treatment of BCR::ABL1-targeting TKIs.

## Methods

### Cell and reagents

K562 cells were purchased from Procell Life Science & Technology Co., Ltd. (Wuhan, China), and were maintained in RPMI 1640 medium (Gibco, Thermo Fisher Scientific) supplemented with 10% fetal bovine serum (Gibco, Thermo Fisher Scientific) in a humidified atmosphere containing 5% CO2 at 37 °C. According to the experimental design, K562 cells were treated with the following reagents: imatinib (MedChemexpress, Monmouth Junction, NJ, USA), nilotinib (Beyotime, Shanghai, China), ponatinib (Beyotime), asciminib (MedChemexpress), MLN4924 (MedChemexpress), TAS4464 (MedChemexpress), VII-31 (MedChemexpress), cisplatin (MedChemexpress), doxorubicin (MedChemexpress).

### CCK8 assay

Four thousand K562 or KU812 cells were seeded into a 96-well plate. Subsequently, 10 µL of CCK8 solution (Beyotime) was added to each well, followed by further incubation at 37 °C for 4 h. The absorbance was measured at 450 nm on a microplate reader (Thermo Fisher Scientific, Waltham, MA, USA).

### Hoechst 33342 live cell staining

Eight thousand K562 cells were seeded into a 12-well plate, 10 µL of Hoechst 33342 live cell staining solution (Beyotime) was added to each well. The stained cells were then photographed under a fluorescence microscope (Olympus, Tokyo, Japan).

### Cell transfection

K562 cells were seeded into a 6-well plate, cell transfection was performed using Hieff Trans^®^ mrna Transfection Reagent (YEASEN Biotech, Shanghai, China). Briefly, 600 µL OPTI-MEM medium was mixed with 1 µg siUBA3 or siNC (General Biol, Chuzhou, China) and 4 µL transfection reagent in 1.5 mL Eppendorf tubes, then incubated at room temperature for 15–20 min. The mixture was added to the culture medium of K562 cells. After 48 to 72 h, cells were harvested and used for the subsequent experiment.

### Cell apoptosis assay

K562 cells were treated with 0.5 µM imatinib in the presence or absence of 25 µM MLN4924 for 24 h. Cell apoptosis was evaluated using Annexin V-APC/PI Apoptosis Kit (Elabscience, Wuhan, China) following the manufacturer’s protocol.

### RNA sequencing

K562 cells were treated with 0.5 µM imatinib with or without 25 µM MLN4924 for 12 h. RNA sequencing was performed by Cosmos Wisdom Biotechnology Co., Ltd (Hangzhou, Zhejiang, China). RNA sequencing data are deposited in Gene Expression Omnibus (GEO) under accession number GSE275701. Raw RNA-seq data were processed using fastp (v0.20.1) to remove adapter sequences and reads with low sequencing quality. The remaining clean reads were aligned to the human genome (hg38) using HISAT2 software (v2.1.0) with default parameter settings. Transcript assembly was performed using StringTie software (v2.0), and expression of transcripts sharing each gene_id was quantified as Transcripts Per Million (TPM). Differential expression analysis was performed using the R package DESeq2. The GO pathway enrichment analyses in the current study were done by R package clusterProfiler.

### Mouse model

4 × 10^6^ K562 cells were subcutaneously inoculated into 6-to-8-week-old, male nude mice (purchased from Shanghai SLAC Laboratory Animal Co. Ltd.) which housed under specific-pathogen free condition. When tumors were palpable, mice were administered with imatinib (100 mg/kg), MLN4924 (50 mg/kg), or a combination of imatinib (100 mg/kg) plus MLN4924 (50 mg/kg). Injections were performed every other day. Tumor size was evaluated using a caliper, tumor volume was calculated using the formula: V = length × width^2^/2. Ten days after administration, mice were euthanized by CO_2_ anesthesia followed by cervical dislocation, tumors were weighed on a digital balance. Animal experiments were approved by the Animal Ethics Committee of Sir Run Run Shaw Hospital, Zhejiang University School of Medicine. The protocols are in accordance with ARRIVE (Animal Research: Reporting of In Vivo Experiments) guidelines and in accordance with relevant guidelines and regulations.

### Co-IP

K562 cells were lysed using IP lysis buffer (Beyotime). Cell lysates were incubated with magnetic beads (Bio-Rad, Hercules, CA, USA) conjugated with an anti-NEDD8 antibody (Abcam, Cambridge, UK) overnight at 4 °C. Thereafter, beads were washed and eluted with 1× sodium dodecyl sulfate loading buffer (Beyotime) for Western blot analysis. Following antibodies were used: anti-ABL1 (Santa Cruz Biotechnology, Dallas, TX, USA), anti-NEDD8 (R&D Systems, Minneapolis, MN, USA).

### Statistics

Statistical analyses were conducted using GraphPad Prism 8.0 Software (Boston, MA, USA). All data are presented as mean ± standard error of the mean. *P* < 0.05 was considered statistically significant.

## Electronic supplementary material

Below is the link to the electronic supplementary material.

Supplementary Material 1

Supplementary Material 2

Supplementary Material 3

## Data Availability

Data are available from the authors upon reasonable request. RNA sequencing data are deposited in Gene Expression Omnibus (GEO) under accession number GSE275701.
