# Supplementary material for: Neddylation status determines the therapeutic sensitivity of tyrosine kinase inhibitors in chronic myeloid leukemia
Source: Sci Rep. 2025 May 30;15:18978. doi: 10.1038/s41598-025-04153-7 (PMC12125173; doi:10.1038/s41598-025-04153-7)
Supplement: Supplementary file 1 — Supplementary Material 1 [file 41598_2025_4153_MOESM1_ESM.docx]

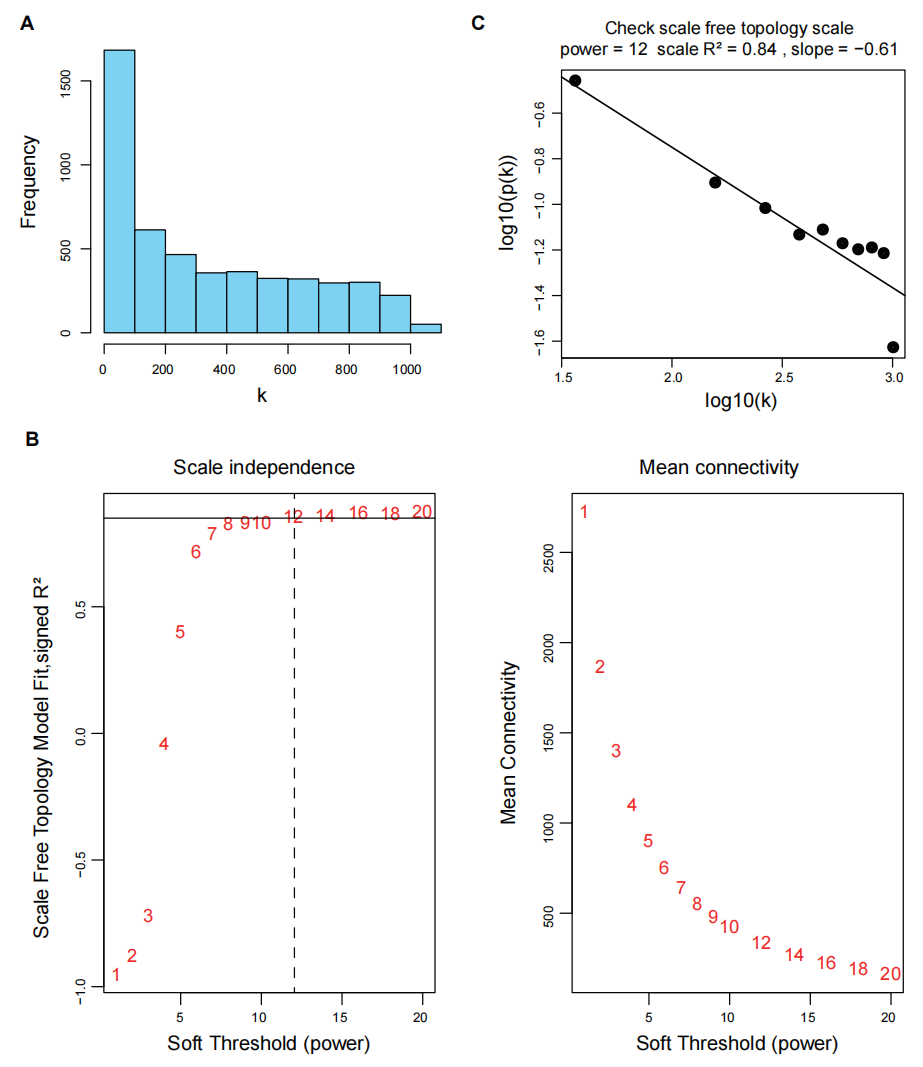


Supplementary figure 1. Weighting coefficient β selection. (A) Bar diagram of scale-free fit index. (B) The verification of scale-free topology with β set as 5. (C) Scatter plot of different soft-thresholding powers with different mean connectivity. Connectivity distribution of nodes with β set as 5.
