# Supplementary material for: Neddylation status determines the therapeutic sensitivity of tyrosine kinase inhibitors in chronic myeloid leukemia
Source: Sci Rep. 2025 May 30;15:18978. doi: 10.1038/s41598-025-04153-7 (PMC12125173; doi:10.1038/s41598-025-04153-7)
Supplement: Supplementary file 2 — Supplementary Material 2 [file 41598_2025_4153_MOESM2_ESM.docx]

**Supplementary table 1. Neddylation prediction for ABL1.**

| Performing IUPred prediction for ABL1_1\|Chain...  Calculating PSSM for ABL1_1\|Chain...  # NeddyPreddy (v0.3) - Prediction Results  # Model Version: 2015-06-25 00:21:10  # Threshold: 0.00 (medium) | | | | |
| --- | --- | --- | --- | --- |
| Identifier | Residue | Class | Decision Value | Probability |
| ABL1_1\|Chain | 15 | Neddylated | 0.36 | 0.18 |
| ABL1_1\|Chain | 84 | Neddylated | 0.39 | 0.19 |
| ABL1_1\|Chain | 87 | Neddylated | 1.15 | 0.48 |
| ABL1_1\|Chain | 508 | Neddylated | 0.00 | 0.10 |
| ABL1_1\|Chain | 593 | Neddylated | 0.19 | 0.14 |
| ABL1_1\|Chain | 605 | Neddylated | 0.80 | 0.33 |
| ABL1_1\|Chain | 606 | Neddylated | 0.11 | 0.12 |
| ABL1_1\|Chain | 658 | Neddylated | 0.10 | 0.12 |
| ABL1_1\|Chain | 711 | Neddylated | 0.35 | 0.18 |
| ABL1_1\|Chain | 727 | Neddylated | 0.07 | 0.11 |
| ABL1_1\|Chain | 756 | Neddylated | 0.98 | 0.40 |
| ABL1_1\|Chain | 788 | Neddylated | 0.44 | 0.20 |
| ABL1_1\|Chain | 816 | Neddylated | 0.30 | 0.16 |
| ABL1_1\|Chain | 879 | Neddylated | 0.10 | 0.12 |
| ABL1_1\|Chain | 909 | Neddylated | 0.16 | 0.13 |
| ABL1_1\|Chain | 913 | Neddylated | 0.43 | 0.20 |
| ABL1_1\|Chain | 957 | Neddylated | 0.26 | 0.15 |
| ABL1_1\|Chain | 1080 | Neddylated | 1.18 | 0.49 |
