# Supplementary figures and images for: Neddylation status determines the therapeutic sensitivity of tyrosine kinase inhibitors in chronic myeloid leukemia
Source: Sci Rep. 2025 May 30;15:18978. doi: 10.1038/s41598-025-04153-7 (PMC12125173; doi:10.1038/s41598-025-04153-7)

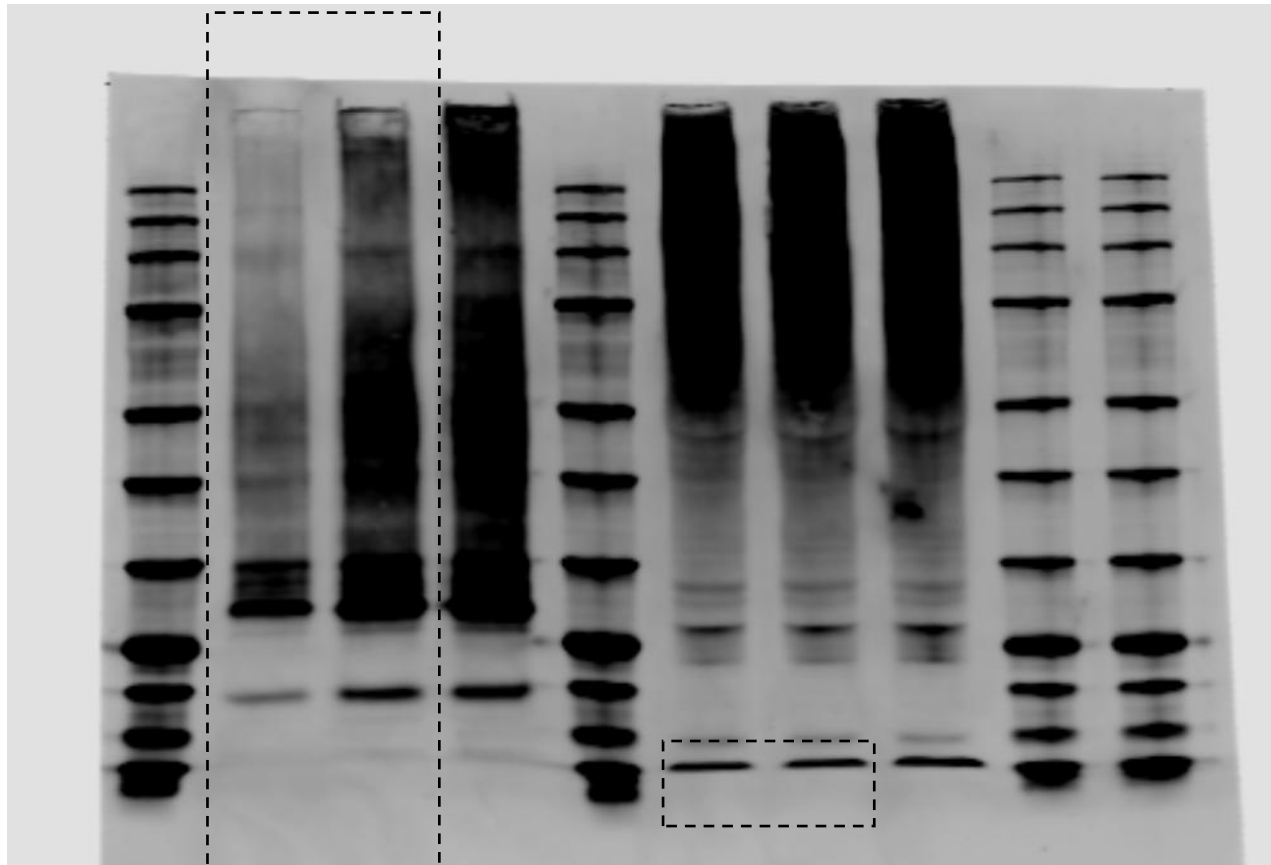

**Figure 4:**  
**IB: NEDD8**

**Figure 4:**  
**Input IB: NEDD8**

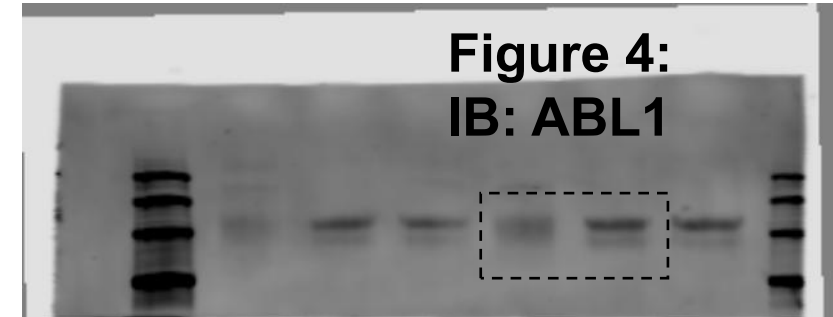

**Figure 4:**  
**IB: ABL1**

Supplement: Supplementary file 3 — Supplementary Material 3 [file 41598_2025_4153_MOESM3_ESM.pdf]
